# Remote Patient Monitoring Using Radio Frequency Identification (RFID) Technology and Machine Learning for Early Detection of Suicidal Behaviour in Mental Health Facilities

**DOI:** 10.3390/s21030776

**Published:** 2021-01-24

**Authors:** Xiaohui Tao, Thanveer Basha Shaik, Niall Higgins, Raj Gururajan, Xujuan Zhou

**Affiliations:** 1 School of Sciences, University of Southern Queensland, Toowoomba 4350, Australia; u1119894@umail.usq.edu.au; 2 Metro North Hospital and Health Service, Royal Brisbane and Women’s Hospital, Herston 4029, Australia; Niall.Higgins@health.qld.gov.au; 3 School of Management and Enterprise, University of Southern Queensland, Springfield 4300, Australia; Raj.Gururajan@usq.edu.au (R.G.); xujuan.zhou@usq.edu.au (X.Z.); 4 School of Nursing, Queensland University of Technology, Brisbane 4000, Australia

**Keywords:** remote patient monitoring (RPM), radio frequency identification (RFID), machine learning, linear regression, decision tree, Random Forest, XGBoost, Ensemble Learning, mental health, suicide

## Abstract

Remote Patient Monitoring (RPM) has gained great popularity with an aim to measure vital signs and gain patient related information in clinics. RPM can be achieved with noninvasive digital technology without hindering a patient’s daily activities and can enhance the efficiency of healthcare delivery in acute clinical settings. In this study, an RPM system was built using radio frequency identification (RFID) technology for early detection of suicidal behaviour in a hospital-based mental health facility. A range of machine learning models such as Linear Regression, Decision Tree, Random Forest, and XGBoost were investigated to help determine the optimum fixed positions of RFID reader–antennas in a simulated hospital ward. Empirical experiments showed that Decision Tree had the best performance compared to Random Forest and XGBoost models. An Ensemble Learning model was also developed, took advantage of these machine learning models based on their individual performance. The research set a path to analyse dynamic moving RFID tags and builds an RPM system to help retrieve patient vital signs such as heart rate, pulse rate, respiration rate and subtle motions to make this research state-of-the-art in terms of managing acute suicidal and self-harm behaviour in a mental health ward.

## 1. Introduction

Healthcare technology has developed rapidly in recent years and innovations in remote monitoring are gaining more attention towards those systems that are capable of early identification of the deteriorating patient [1]. Remote patient monitoring (RPM) is capable of obtaining continuous accurate readings of vital signs and a range of other clinically important information that can identify early indicators of deterioration in a patient’s health condition [2]. This can be achieved with noninvasive digital technology without hindering a patients’ daily activities, and it can enhance the efficiency of healthcare delivery in acute clinical settings. In mental health, this can provide a particular advantage as inpatients do not spend the majority of their time in bed and do not routinely have a clinical monitor or device attached to them for providing treatment and care.

The goal of inpatient psychiatric care is to provide a safe environment for both patients and staff. Strategies to manage or minimise self-harm in depressed and suicidal patients include a range of non-pharmacological approaches such as visual observations and therapeutic engagement. However, when a patient has persistent suicidal ideation they are in a very vulnerable state and have a high risk of acting upon this particularly in the early stages of admission [3]. In-hospital suicides are also often associated with occurring in the evening and during night shifts when there is reduced staff supervision. During these times of high risk, suicides occur in isolated areas of the ward such as bathrooms and single rooms. RPM technology has the potential to assist with identifying early signs of suicidal intent before these occasions occur and would be a valuable clinical tool for effective management in providing care [1].

Current technological advances in remote patient monitoring can record clinical observations related to varying physical phenomena. Continuous monitoring of human vital signs such as heart rate, pulse rate, breathing rates and patient movements could act as important clinical indicators for early detection of deterioration [4]. Alterations in any of these parameters could be used to predict serious clinical events [5]. The clinical utility of this system lies with routine visual observations and patient safety monitoring by nursing staff. Staff could be alerted by an RPM system to return to a patient room and assess the safety of this patient if they have left their bed shortly after being observed to apparently be sleeping. Other recent technologies on Internet of Things (IoT), including Heterogeneous Brain Storming (HBS) for object recognition tasks [6], also have great potential to advance remote patient monitoring.

There are known benefits to using radio frequency identification (RFID) technology in healthcare asset tracking and predicting future events with machine learning tools by analysing RFID tag data [4]. These techniques can also be used for direct patient care by providing alerts to indicate potential human errors in managing patients such as misidentification of patients and medication [7]. However, traditional RPM systems are intrusive and need dedicated sensors to be attached and can cause considerable inconvenience in psychiatric clinical care. Recent innovations have employed this technology to detect vital signs without making contact with the patient using techniques such as near-field coherent sensing of signals generated from the antennae of the RFID tag [8,9]. However, little is known about how these methods can be used to monitor vital signs of several patients simultaneously who are also mobile. There are formidable challenges with integrating this type of technology into a clinical setting such as where best to deploy equipment for it not to interfere with routine clinical care.

The ultimate goal of this research is to detect accurate vital signs of multiple patients through an RPM system. Readings for each individual will be tracked and differentiated using noninvasive monitoring techniques [7]. Identifying the optimum configuration of sensors using RFID technology in a simulated hospital ward has become an important step toward this goal. In this study, a laboratory was established, simulating a hospital psychiatric ward using an RPM system utilising sensors and RFID technology. We collected and analysed data from multiple reader–antenna positions in the laboratory. Specifically, we compared combinations of multiple positions of reader–antennas based on received signal strength indicators (RSSI) and from this derived better positions to fix the reader–antenna devices. The linear relationship between dependent and independent variables was derived with their coefficients and an intercept with using an ordinary least squares (OLS) linear regression method. A linear equation was developed with RSSI as output variable and *Distance*_1, *Distance*_2, *Antennas*_*Distance* as independent variables with their respective coefficients. Label prediction was completed using various machine learning algorithms and derived their error metrics, aiming to discover the patterns between the positions of reader–antenna and RSSI readings. As a result, Decision Tree appeared to have the best performance compared with Random Forest and XGBoost. Furthermore, an Ensemble Learning model was developed, capitalising on the performance advantages of these machine learning algorithms. This study contributes towards better design of a remote patient monitoring (RPM) system with noninvasive technology, specifically:

- better understanding of methodology and complications involved to kick-start data collection of human vital signs and their motion;
- setting reader–antennas with efficacy to have high tag readability in a simulated psychiatric ward;
- discovered the patterns between independent and dependent variables retrieved from RFID equipment setup on a static tag;
- better understanding of a number of existing machine learning algorithms, specifically, Decision Tree, Random Forest and XGBoost, for their capability of data analytic in an RPM system utilising sensors and RFID technology; and furthermore
- an ensemble machine learning model developed for data analytic in an RPM system utilising sensors and RFID technology.

In this paper, Section 2 presents a review on existing methods in monitoring depressed patients and RFID implementation in Remote Patient Monitoring systems. In Section 3, the research problem will be defined, and in Section 4, the research methodology will be discussed, including the technical details of data collection and data modelling. The experimental results are presented and discussed in Section 5. Finally, Section 6 concludes the paper with research outcomes, contributions and limitations.

## 2. Related Works

Healthcare-related RFID technology for patient care has previously been implemented using passive RFID tags that have been integrated with hospital identification bands worn on the patient’s wrist. These typically have been useful for retrieving patient details like name, age, blood type, treatments required, allergies and so on by scanning an RFID tag with a reader [7]. This has been shown to improve hospital safety measures by working as a smart identification system [4,10]. However, a limitation of this is in situations where the patient does not wear a wrist band [11]. This is common in psychiatric wards where identification of patients is routinely performed using a photographic system [12]. A recent attempt to overcome this constraint has had modest success where psychiatric nurses conducted visual observations via infrared based sensors fitted in a fixed installation located within an anti-ligature housing on the wall.

Wang et al. [13] described a case study demonstrating how an RPM system using RFID technology was implemented hospital-wide in the Taipei Medical University Hospital around 14 years ago amid the SARS outbreak. The authors described the development strategy and architecture design, and highlighted the importance of support from clinical staff for successful implementation of an RPM system in a hospital. This innovative project included a purpose specific active RFID tag that was developed to monitor patient temperature. Field generators were used in conjunction with tag readers to help contain costs. The readers had multiple read capacity with 10 MB memory and a range of 3 to 85 metres. The architecture developed for the location-based management system was sophisticated and could retrieve data from a medical record system before processing and sending an alert/email/short message to clinical staff when it judged there was an infectious event. The system included readers outside the hospital that were connected to the local telecommunication networks to assist with community quarantines.

Researchers at Cornell University have recently developed a method of detecting vital signs using Near-field Coherent Sensing (NCS) based on electromagnetic energy [14]. This is achieved by indirectly modulating the backscattered signal generated from mechanical motions that originate from inside the human body as well as on the surface. The signal source from the pulse should be within the near-field zone of the antennas, around 10 cm, with amplitude and phase used for measurements. The NCS method overcomes the limitations of existing systems like electrocardiogram and acoustics methods that require direct contact with skin and are also limited by body movement, patient comfort and wearing convenience [15]. NCS also has advantages over other methods such as photoplethsmography that are dependent on reflection and transmission of light, which can limit sensing capabilities and sampling rates thereby compromising the accuracy of monitored heart rate and respiration rate [8].

An RPM system using NCS with RFID technology was described by Sharma and Kan for the purpose of sleep monitoring as a viable alternative to polysomnography [16]. The system was implemented using passive RFID tags to assess heart rate, respiration rate and upper body movement. The RFID tag was adhered to the fabric of the patients clothing near the region of the heart. Semi-supervised learning of temporal and spectral characteristics for classification by support vector machines was completed using signal features when the patient was at rest, with accurately detected motion in 91.06% of cases. Another study that assessed patient movement through an RPM system was in an Australian hospital that used passive RFID tags on patient clothing [17]. Identification of movement was based upon the backscattered signal at the RFID reader antenna. A wearable sensor system was developed to detect patient movement when getting out of bed or a chair to prevent falls and was able to detect these motions 81.4% of the time. Zhao et al. also designed an RPM system to detect movement of people by measuring signal changes of pre-deployed passive RFID tags at critical places [18]. The system estimated moving direction and current location by measuring critical power variation sequences of the passive RFID tags.

The goal of this work is to design an RPM system that can be readily transferred from the lab environment for monitoring in an acute care setting. To achieve the goal of a real-time monitor for vital signs and movements of acutely suicidal patients in a real hospital ward, an initial step was to understand the optimum configuration of reader–antenna positions in a simulated hospital ward. Radio wave signals need to be detected and transmitted from passive RFID tags. As discussed above, many methods in related studies to date have relied upon manually collected data. Although promising, the deployment of these processes in a real clinical environment would be time consuming and error prone with much manual effort required. Studies that incorporate remote monitoring with RFID technology appeared to be more feasible for detecting patient motion and monitoring vital signs under real clinical conditions.

## 3. Research Problem

The research problem is to determine the optimum configuration of an RPM system in a simulated hospital ward that can effectively monitor vital signs and patient movement. The study was divided into two stages: The first was to determine the optimum position placement of two reader–antennas from a range of different combinations that could effectively read data signals generated from a static passive RFID tag and to feed signal data into multiple machine learning models so that a dependent variable could be predicted. This was based up on comparisons of the various received signal strength indicators (RSSI). The second stage was to determine the error rate in predicting the dependent variable by evaluating the performance of the regression models produced using schemes commonly recognised by the research community in this field.

## 4. Methodology

### 4.1. Framework

The study was conducted in a dedicated lab room to simulate a hospital ward for real-time human data collection and build machine learning models to analyse the data patterns. To detect the position of a passive RFID tag placed in the room, a setup was needed with multiple reader–antennas. Two ultra-high frequency (UHF) 870 readers with integrated antennas were chosen to detect the RFID signal data. Initial preparatory work included an analysis of the radiation pattern generated from the readers. Polarity of the reader was taken into consideration and its orientation in relation to the tag was chosen based on minimising the effect that this had on signal detection.

RFID reader–antenna positions were tested by collecting data from the static tag to establish the optimum position of each of the antennae where maximum signal strength was received. Except from reader–antennas, RFID passive tag and the lab computer, the laboratory room was free from any other objects to ensure measurement accuracy. The retrieved tag data was read using the manufacturer software and UHF reader utility that was installed on a lab computer and preprocessed to compare signal strengths of the two reader–antennas. The relationship between the input and output variables was thus based on the distance between the passive RFID tag and the antennae of the two readers. The RSSI was chosen as the dependent output variable. The two measured distances of the readers from the static tag were the independent variables. The distance between the two reader–antenna positions was also measured and regarded as independent variables in the data set. The linear relationship between the variables, coefficients and the intercept was calculated using ordinary least squares (OLS) linear regression and is shown in Equation (1).

(1) $$f(y)=\left(m_{1}\times x_{1}\right)+\left(m_{2}\times x_{2}\right)+\cdots +\left(m_{n}\times x_{n}\right)+c$$

where f(y) is the output variable with $m_{1},m_{2},\cdots ,m_{n}$ as coefficient of input variables $x_{1},x_{2},\cdots ,x_{n}$, taking *c* as the intercept of the line equation.

**Figure 1:**
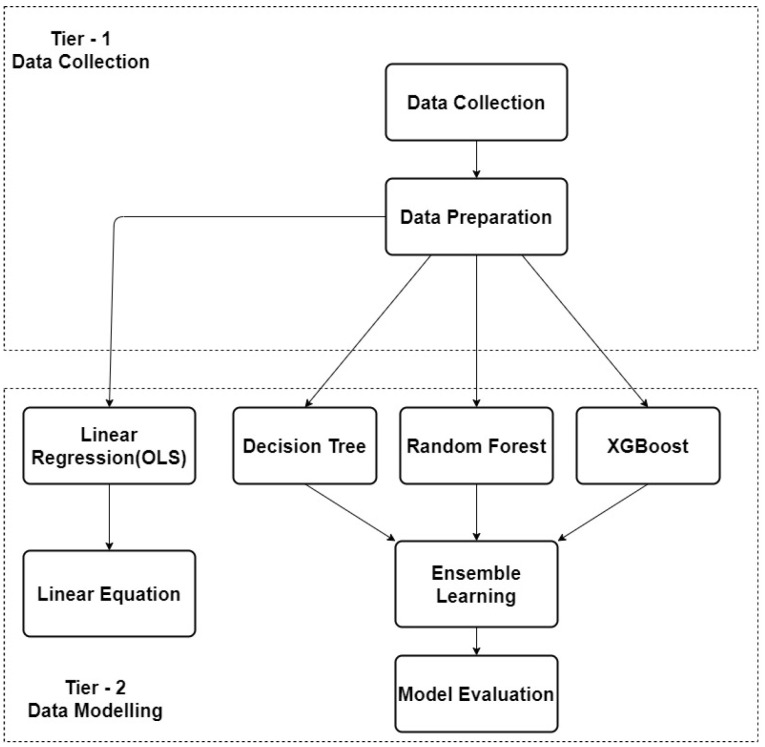
Overview of research architecture.

**Figure 2:**
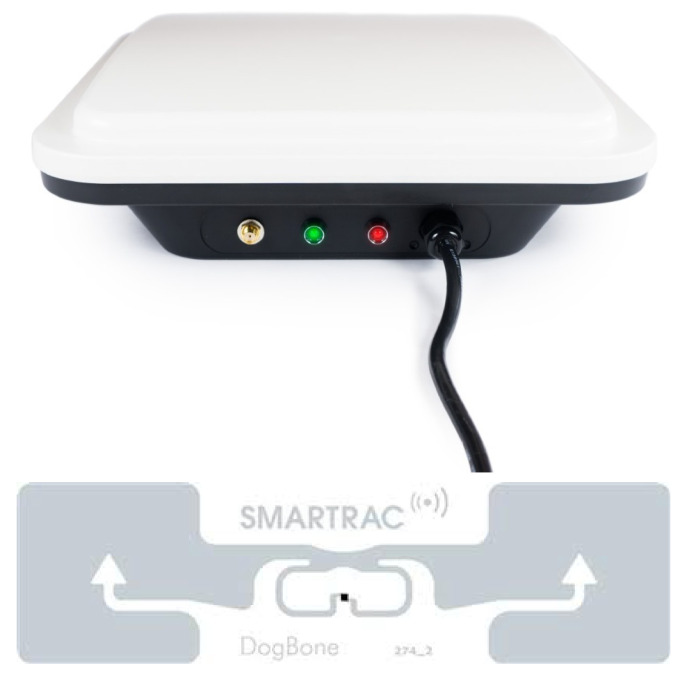
Ultra-high frequency (UHF) radio frequency identification (RFID) reader–antenna and passive RFID tag.

The architecture of this study is illustrated in Figure 1, which uses a framework with two tiers, separating the data collection and data modelling aspects. Data collection included the receivable signal strength on two UHF 870 RFID reader–antennas (shown in Figure 2) positioned in 16 arbitrary positions in the laboratory to test reader–antenna signal receivable strength. Two UHF 870 RFID reader–antennas were placed in selected positions in the simulated ward that received data from the static tag. As previously stated, the research problem is to determine the multiple reader–antenna positions in the laboratory/simulated ward, RFID passive tag position which was made static. All the coordinates of the various reader–antenna positions and including the tag were recorded. Later, the data set was preprocessed in a data preparation step. The recorded coordinates and of reader–antenna positions and tag were used to calculate the distances between the tag and each of the reader–antenna positions.

Data modelling included first a linear equation, which was derived from the relationship between the chosen variables using Ordinary Least Squares (OLS). Machine learning algorithms were then subsequently chosen for modelling and data analysis, aiming to detect this linear relationship and to predict label values. An Ensemble Learning method was developed, taking advantage of these models for further performance improvement.

### 4.2. Data Collection and Preparation

#### 4.2.1. Sensor Technology adapted to a Simulated Ward

**Figure 3:**
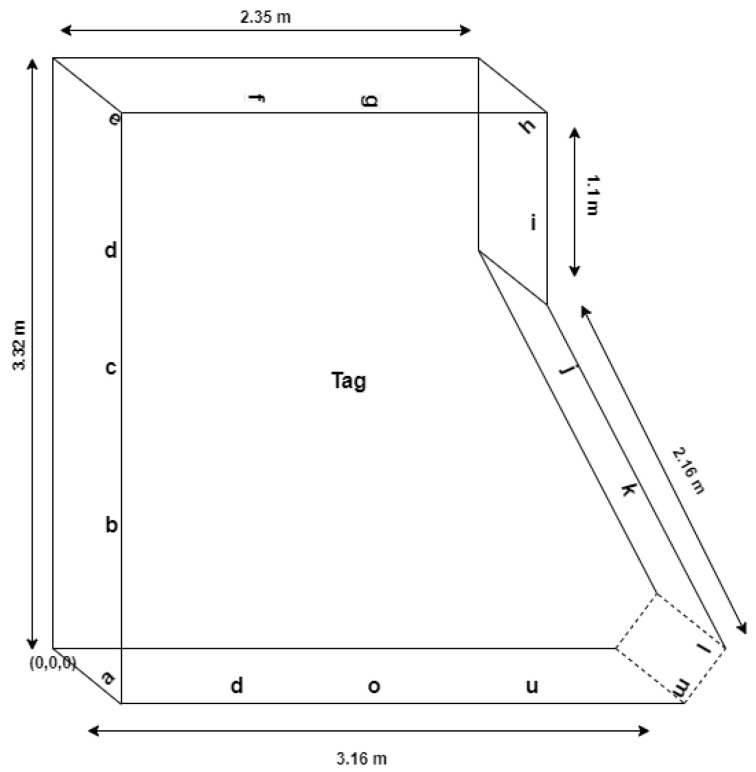
Laboratory room/Simulated ward with reader–antennas and tag positions.

The two UHF RFID reader–antennas that were used in the study together with a passive RFID tags are illustrated in Figure 2. A standard laboratory computer was used to operate reader–antennas through manufacturer software and to read reader–antenna retrieved tag data. Reader–antennas were fixed to side walls of the simulated ward shown in Figure 3 and the RFID tag placed at centre of the room fixed to a stable object, simulating a patient lying on a hospital bad. The dimensions of the simulated ward indicate where the alphabetically represented reader–antennas were positioned. The first reader–antenna was initially placed at position *a*, and data were recorded from both readers where each time the second reader–antenna was sequentially placed at each of the other 15 identified reader–antenna positions from *b* to *p*. Reader 1 was then placed in position *b*, and the corresponding Reader 2 moved accordingly to each of the remaining 14 positions from *c* to *p*. This pattern of moving the readers continued until all combinations were completed. Table 1 shows the *x*, *y*, *z* coordinates of the chosen reader–antenna positions measured in metres with the original point (0,0,0) at the lower-left corner of the room.

#### 4.2.2. Radiation Pattern

The high gain directional antennas of the UHF RFID readers concentrated the RF field in a specific direction. As the RFID passive tags do not have an internal power source electromagnetic energy transmitted from the RFID readers activated the tags. The Friis transmission formula shown in Equation (2) was applied to manage the power received by the passive tag that was transmitted from the RFID readers [19]:

(2) $$\frac{P_{tag}}{P_{reader}}=G_{tag}G_{reader}{\left(\frac{\lambda }{4\pi d}\right)}^{2}$$

where $P_{tag}$ and $P_{reader}$ are power received by the tag and power transmitted by reader, respectively. $G_{tag}$ and $G_{reader}$ are the corresponding gains of the tag and reader antennas, λ is wavelength and *d* the distance between tag and reader antenna.

High directional gain antennas have an oval radiation pattern and read the tags in the peripheral area. The antenna radiated a very narrow beam over a long distance and used point-to-point communication. The top side of the reader–antenna devices were positioned so that they faced towards the static tag and tilted slightly downwards. This was to accommodate the oval radiation pattern and the location of the static tag that was lower than the arbitrarily chosen positions of the reader–antennas.

#### 4.2.3. Reader–Antenna Polarisation and Orientation

Polarisation is orientation of the electric field of an electromagnetic wave. Linear polarisation and circular polarisation are special cases of elliptical polarisation. With linear polarisation, the electric field stays in same plane [20]. Therefore, reader–antennas and the RFID tag were located within the same plane to minimise the possibility of orientation mismatches [21].

RFID tags are usually matched with linearly polarised antenna with either a horizontal or vertical orientation. However, the aim here is to detect tags that may be in any orientation and capable of detecting vital signs and movements of a hospitalised patient. The lab was therefore constructed using circularly polarised antenna. Although a limitation of this technique is that the read range is much shorter, it does have the ability to identify an RFID tag irrespective of orientation.

#### 4.2.4. UHF Reader Utility Software

Prior to data collection several parameters of the UHF reader utility software including RFID tags used in the experiments were configured. These included reader frequency (918–926 MHz for Australia), tag alias name and Auto-Save path for retrieved data. Additional attributes included Received Signal Strength Indicator (RSSI) and frequency of the tag. As both UHF reader–antennas were set to the same configuration and features, it was expected that the second antenna position would have the same distribution of data. Reader operation mode was set to Auto-Read for reading tags that were in the reader–antenna radiation pattern. This was user-dependent and needed to be reset if a reader did not detect a tag within a given time frame. Data collected from successful tag readings were saved in a csv format file by the Auto-Save feature.

#### 4.2.5. Data Preparation

Data were collected on a csv sheet for each of the chosen 16 paired reader-antenna positions. The relationships between reader–antenna position and the corresponding received signal strength were analysed based upon the seven features listed below.

- *First Antenna Position*: alphabetic notation.
- *Distance 1*: distance of tag from first antenna in metres.
- *Frequency 1*: frequency of the tag w.r.t Antenna 1 in MHz.
- *RSSI 1*: Received Signal Strength Indicator (RSSI) percentage w.r.t Antenna 1.
- *Second Antenna Position*: alphabetic notation.
- *Distance 2*: distance of tag from second antenna in metres.
- *Frequency 2*: frequency of the tag w.r.t Antenna 2 in MHz.
- *RSSI 2*: Received Signal Strength Indicator (RSSI) percentage w.r.t Antenna 2.

Collected data was preprocessed prior to training the machine learning models. Data features from the two reader–antennas described above were combined to make the data tag-centric. From this the following six features were recorded based upon taking the maximum value from either RSSI 1 or RSSI 2.

- *First Antenna Position*: alphabetic notation.
- *Distance 1*: distance of tag from first antenna.
- *Second Antenna Position*: alphabetic notation.
- *Distance 2*: distance of tag from second antenna.
- *Frequency*: frequency of the tag.
- *RSSI*: Received Signal Strength Indicator (RSSI).

**Figure 4:**
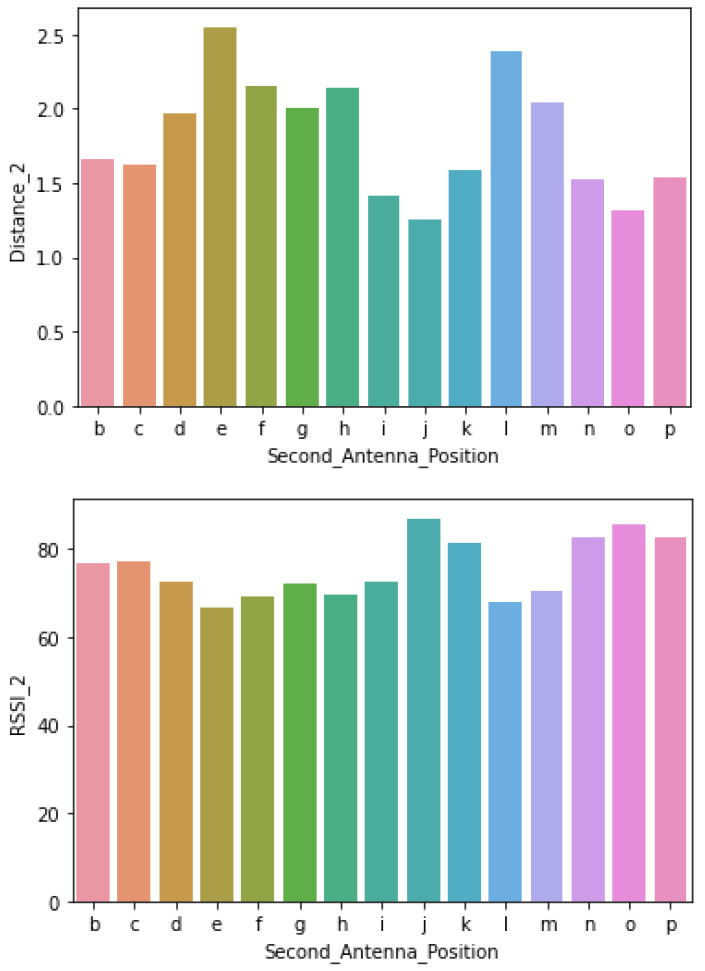
Second Antenna position distance and Received Signal Strength Indicator (RSSI), where the top is reader–antenna position and its distance from tag, and the bottom is reader–antenna position and RSSI.

The independent features were *Distance 1*, *Distance 2* and *Antenna Distance*, whereas the *RSSI* was the dependent feature. Their relationship can be revealed from the plots on Figure 4, where shorter distances correspond to stronger RSSI and vise versa. Detailed statistics of the collected data set are presented in Section 5.

### 4.3. Data Modelling

In the second tier, Data Modelling, machine learning algorithms were executed for data modelling and analysis. The relationship between the independent and dependent variables was determined using ordinary least squares (OLS) method in linear regression model. Later, machine learning algorithms such as Decision Tree, Random Forest and XGBoost were adopted to predict signal strength received by reader–antenna in various positions. Finally, an Ensemble Learning was developed to combine the individual machine learning models, aiming to improve predictive performance of the model.

#### 4.3.1. Relationship between Variables

Ordinary Least Squares (OLS) was the statistical method used to define significance of each independent feature in the data set with respect to dependent features [22]. The collected data were split into training data and testing data on a 4:1 ratio to perform the OLS regression. Using this method the sum of square differences between the variables were minimised before deriving the linear equation shown in Equation (1). We also calculated an *r*-squared, adjusted *r*-squared and a *p* value to show significance of the independent features and their coefficients relative to each of the independent features. The related analysis is discussed in Section 5.

#### 4.3.2. Signal Strength Prediction

Machine learning algorithms were adopted to model the patterns between the strength of received signal and the position of reader–antennas. Using the collected data set without additional parameter tuning, a preliminary empirical study was conducted, aiming to find out the right machine learning algorithms for data modelling in this context (The preliminary empirical experiment was conducted on the *Lazy Predict* package in the library of Python version 3.8). As shown in Table 2, a large number (35) of existing machine learning models were tested in the study. Their performance was measured by *r*-squared values and mean square error (MSE; see Equation (6) in Section 5.1).

Based on the empirical experimental results shown in Table 2, Decision Tree, Random Forest and XGBoost were identified as the top three models and were thus selected for data modelling in this study. Their technical details are discussed below.

**Decision** **Tree** is a predictive modelling approach commonly used in statistics and machine learning. As the target variable in this study was continuous, we used the regression tree function to predict our output dependent variable. To assist with modelling in Decision Tree, we set *max_depth*, the maximum depth parameter in the experimental system (The *Sklearn* package in the library of Python version 3.8) to determine the furthest extent of the decision that predicted the label value. The maximum depth parameter was obtained using testing values between 1 and 10. The results show that when the maximum depth parameter is 8, the model has the best performance—least mean absolute error (MAE; see Equation (5) in Section 5.1) and mean squared error (MSE). Decision tree was fit with the specific data set to train the model.
**Random** **Forest** is a type of supervised machine learning model based on Ensemble Learning by combining multiple models to form a more accurate prediction model. The regression model was used to aggregate the Decision Tree predictions and produce a meta-estimation of the target variables. Each decision tree predicts a target variable for each record and then the average of all decision trees is taken to predict the final output. The number of trees chosen for the Random Forest was based on trial and error with different values to tune the *n_estimator* hyperparameter. This was not time consuming as Random Forest is not computationally expensive. There were 10 trees eventually chosen as the *n_estimator* for the forest to enhance prediction capability of the model and reduce error rates. The *max_depth* parameter remained the same as that chosen for the Decision Tree model. To measure impurity of the output variable, mean squared error (MSE) was again used.
**XGBoost**  The extreme gradient boosting technique known as XGBoost was also used to build the trees [23], and it is highly flexible, efficient and portable. The modelling was completed in a sequential fashion with the aim of reducing errors in previous trees from the results of subsequent trees. The model not only learns from predecessors but also optimises residual errors in each tree by using a gradient descent to minimise error iteratively. Aiming to better train the model, the data set was split using *k*-fold cross-validation with 9 sets for training and one for evaluation. The standard MAE and MSE performance metrics for this study were again calculated. MSE also provided the loss function to estimate training loss. The objective function in Equation (3) was estimated with this training loss together with a regularisation term to measure how well the model fitted the training data.
  (3) obj(θ)=L(θ)+Ω(θ)
  where *L* is the training loss function and Ω the regularisation term.
**Ensemble** **Learning** The current study is limited by the number of chosen positions in which the receiver–antennas have been placed. The expected data set from 16 positions is relatively small, so it was assumed that the hypotheses produced from the chosen machine learning algorithms would be prone to overfitting thus affecting the quality of their predictive ability. An Ensemble Learning algorithm was therefore applied to improve the predictive performance of the final model [24]. The application of Ensemble Learning averaged the different hypothesis predicted from the previously applied algorithms and reduced the risk of selecting an incorrect hypothesis [25]. The weighted average ensemble model shown in Equation (4) combines outcomes from the Decision Tree, Random Forest and XGBoost models.
  (4) $$ensemble\_model=\sum_{i=1}^{n}\left(w_{i}\times m_{i}\right),$$
  where $m_{i}$ refers to a model weighted by $w_{i}$, *n* is the number of available models (which is 3 in this case), and $w_{1}+\cdots +w_{n}=1$.
  Each model input to the ensemble was weighted with a coefficient valued between 0.1 to 1.0. The coefficients were iteratively balanced to achieve a combined weight equal to 1.0. Similar performance metrics were again calculated from this resultant estimated average of the three previous model predictions.

## 5. Experimental Results and Analysis

### 5.1. Performance Metrics

Two different performance metrics were used to evaluate the regression models implemented: mean absolute error (MAE) and mean squared error (MSE).

MAE provided a measure of the average magnitude of errors in predictions excluding direction. This value ranges from 0 to 1 and was negatively-oriented so that lower values produced from Equation (5) indicated a better model. The following metrics were used to evaluate each regression model including weighted average output.

(5) $$\mathrm{MAE}=\frac{\sum_{i=1}^{n}\left|y_{i}-x_{i}\right|}{n},$$

where $y_{i}$ is the predicted value, $x_{i}$ the actual value and *n* the total number of data points.

Mean squared error (MSE) measures average of the squared error. It is calculated by summing squares of the difference between the predicted values and actual value, divided by number of data points. Equation (6) shows how the MSE was calculated for measuring impurity:

(6) $$\mathrm{MSE}=\frac{1}{n}\sum_{i=1}^{n}{\left(Y_{i}-\hat{Y_{i}}\right)}^{2}.$$

MSE is the mean $\left(\frac{1}{n}\sum_{i=1}^{n}\right)$ of the squares of the errors ${\left(Y_{i}-\hat{Y_{i}}\right)}^{2}$.

### 5.2. Experimental Results

Reader–antenna positions within the simulated ward were recorded based upon their coordinates. The 16 arbitrarily chosen reader–antenna positions resulted in 120 combinations of the paired readers. General descriptive statistics of the data collected are presented in Table 3. This includes the number of records collected from the positions selected for analysis, mean and standard deviation of the variables, minimum and maximum values together with quartile ranges. The range of each feature differs as the measuring unit for each one is different. All features have the same number of records with no missing values. Table 4 presents the top 5 records of the data set features after data preparation was completed.

**Figure 5:**
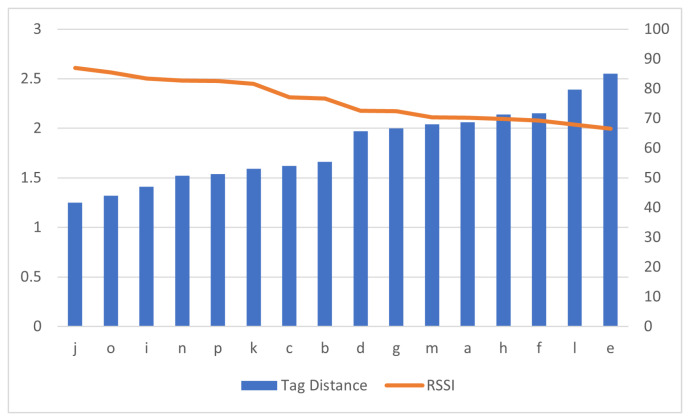
Fall in RSSI with tag distance.

Prior to data preparation, the RSSI values of all reader–antenna positions in the simulated ward were plotted on a line graph with respect to a bar graph of the static tag position and are presented in Figure 5. The alphabetic positions of the reader–antennas are denoted on the *x* axis and the static tag distance in meters is on the $y_{1}$ axis. The RSSI percentage value is denoted on the $y_{2}$ axis ranges from 0 to 100. The maximum RSSI value was approximately 86% and steadily decreased to 66% as the readers were brought further apart. The closest antenna position on the bar graph is the *j* position and farthest is the *e* position.

Data collected from the paired reader positions were analysed. Table 5 shows the matrix formed. The maximum RSSI value gathered from the two reader–antennas is presented. Each of the 16 reader–antennas was combined with the remaining reader–antenna positions. There were no repetitions as both reader–antennas were identical in both configuration and features. Close observation of Table 5 showed that *j* antenna position had the highest RSSI value and *e* position had the least RSSI value.

Using the ordinary least squares (OLS) linear regression method, a linear relationship between independent and dependent variables was determined. The OLS results are presented in Table 6. It shows that the two independent variables, *Distance*_1 and *Distance*_2, have a negative coefficient. *Antennas*_*Distance* variable has coefficient closer to zero value. The constant value was the intercept of the linear equation. Standard error is error in prediction or represents the average distance of the variable from the regression line. Standard error was high in constant value compared to other three variables. The *t*-statistic value is a measure of how statistically significant the coefficient is, which was calculated by dividing coefficient with standard error. The constant value had a high *t*-statistic value due to its high standard error.

The statistical significance of each variable, which effects the output variable RSSI value, is tested in this OLS linear regression analysis. The p>|t| value as shown in Table 6 defines the significance of the variables, which is the *p*-value for the null hypothesis that the coefficient is equal to zero (no effect). By convention, α value (0.05) was set to be the standard measure for significance. If *p* value of a variable was less than α, the variable was considered to be statistically significant. In this study, *Distance*_1 and *Distance*_2 variables were highly significant and the null hypothesis was rejected, i.e., distance of static tag from each antenna–readers would not affect the RSSI value as their *p* value was much less than the α value. This showed that the *Distance*_1 and *Distance*_2 variables were strongly correlated with dependent variable RSSI and a value decrease in these variables would enhance the output variable. *Antennas*_*Distance* variable’s *p* value was also greater than the α value 0.05 and did not reject the null hypothesis. The *r*-squared value is a fraction of variation in output variable predicted by input variable [26]. Here, the value is 0.626.

From Table 6, a linear Equation (7) can be formed with coefficients of each variable and constant value.

(7) $$f(y)=109.22+\left(-6.01\right)\times v_{1}+\left(-11.05\right)\times v_{2}+\left(0.27\right)\times v_{3},$$

where f(y) is the output variable, with input variables *Distance*_1, *Distance*_2 and *Distance*_3 being $v_{1}$, $v_{2}$ and $v_{3}$, respectively, and the constant is the intercept.

As part of the data modelling tier of this study architecture, multiple machine learning models, such as Decision Tree, Random Forest and XGBoost, were trained and evaluated with performance metrics discussed in Section 5.1. The decision tree model had performance metrics as MAE 0.01 and MSE 0.003. The hyperparameter of Decision Tree, max_depth, was tuned from 1 to 10. This reduced the error rates till the value 8. Random Forest had a higher performance compared to XGBoost with MAE 0.16 and MSE 0.11. Two hyperparameters—n_estimators and max_depth—of the Random Forest model were tuned to enhance the prediction capability of the model and reduce the error rates. In the XGBoost model, the error rates are high compared to the other two machine learning models. The *k*-fold cross-validation implemented in the XGBoost model did not seem to improve the performance. Later, Ensemble Learning was implemented by combining individual models with weights, taking average of the outputs as the final result. Decision Tree had the least error rate in all three performance metrics with MAE 0.01 and MSE 0.003, compared to other individual models, even with outperforming the Ensemble Learning model. Performance metrics of the individual models and Ensemble Learning are presented in Table 7.

Ideally, Ensemble Learning would enhance the prediction accuracy compared to individual models involved in it. In this study, the weighted average method did not improve prediction accuracy or reduce error rates, at least when compared with the Decision Tree model. After tuning the model by testing the coefficients in range of 0.1 to 1, the best performance that the Ensemble Learning model was able to achieve was MAE 0.04 and MSE 0.006 (as shown in Table 5) with $w_{1}=0.8,w_{2}=0.1$ and $w_{3}=0.1$, where $m_{1},m_{2}$ and $m_{3}$ refer to Decision Tree, Random Forest and XGBoost models, respectively, for the ensemble_model function defined by Equation (4).

**Figure 6:**
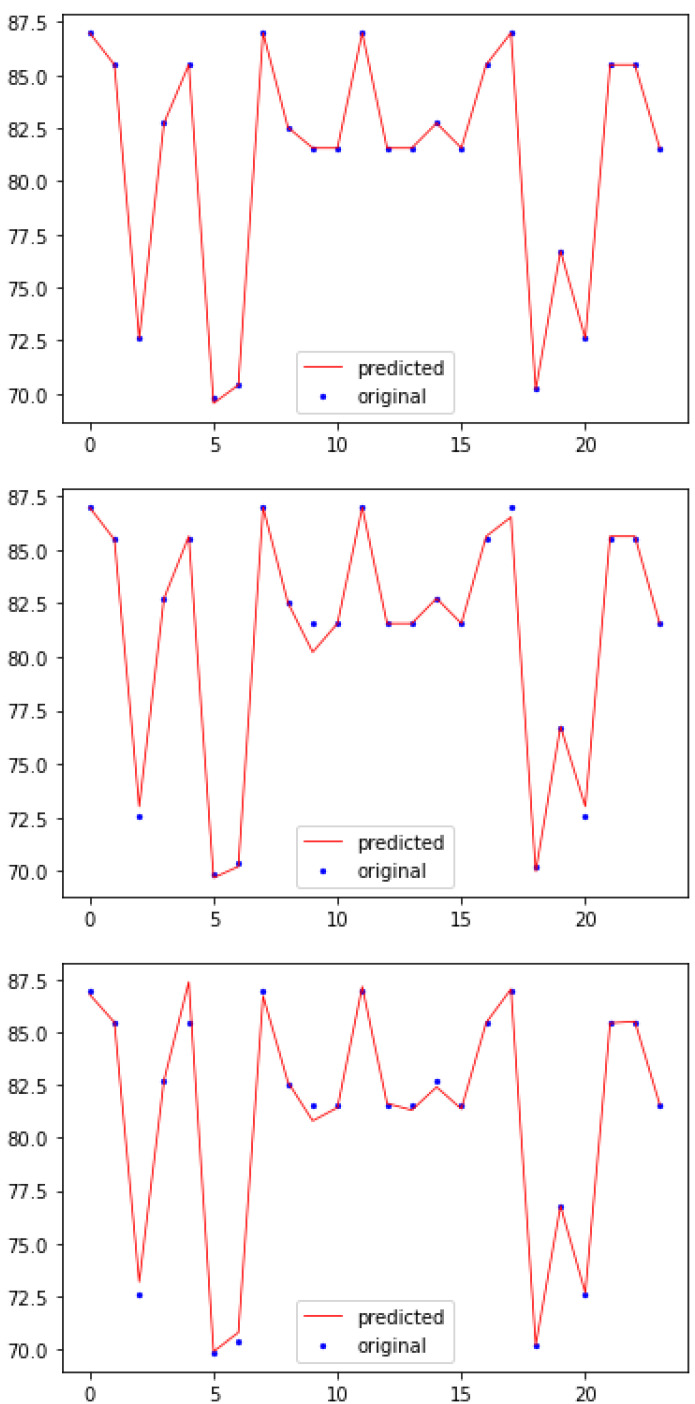
Models evaluation plots with Decision Tree sitting on top, Random Forest in the middle and XGBoost on the bottom.

The predicted values of test data were compared with original test data to evaluate the individual model’s predictive performance visually. Figure 6 shows three plots of Decision Tree, Random Forest and XGBoost models. To differentiate the original data and predicted data in the plots, dots refer to original data and lines refer to predicted data. If a line overlaps a dot, the model has predicted the value precisely; otherwise the model has predicted incorrectly. Fluctuations in the original and predicted data seem almost similar in three model plots. Decision Tree model was able to predict almost all data points precisely. Random Forest plot shows four data points were incorrectly predicted, whereas XGBoost model predicted five data points incorrectly. This is consistent with the performance metrics shown in Table 7.

### 5.3. Discussion

The main contribution that this study makes is to the understanding of the ground work required for designing a remote patient monitoring system using RFID sensor technology. This work has identified the considerations needed for using RFID reader–antennas to identify vital signs on hospitalised patients that may be able to move freely about the ward. The work implies that this type of approach would be best deployed in patient rooms that are designed to accommodate possibly up to four in-patients instead of an open ward layout. This is because the range for detecting passive RFID signals using the techniques described in this study have a direct bearing on the RSSI. However, this conforms to the goal of the initial scenario for this research, which is to identify early deterioration of suicidal and self-harm behaviours in circumstances where nursing observation and supervision of patient safety are reduced because of lower staffing levels during the known high risk periods in the evening and night shifts. The study has confirmed how the optimum positions for reader–antennas would be chosen for deployment in a psychiatric ward of a given hospital. This was achieved by understanding the relationship between the independent and dependent variables that contribute to detecting the maximum signal strength required for detecting vital signs and patient movement. A better understanding was also offered from the results of this case study as to the implications for choice of a suitable machine learning algorithm to analyse signal data.

The optimum position for the first reader–antenna placed in the simulated ward used for this study produced the highest RSSI tag readability. The second reader position was determined from the next highest signal strength obtained. A benefit of this for eventual system design is that the location of at least one of the reader–antennas could logically be associated with an entrance or doorway and could indicate if a person has left the room with respect to the signal strength associated with this event. The impact of this relationship to tag readability has important practical implications for deployment in a real ward and could effect decisions on how notifications and alarms will be configured so not to disrupt the routine clinical business of a hospital ward.

**Figure 7:**
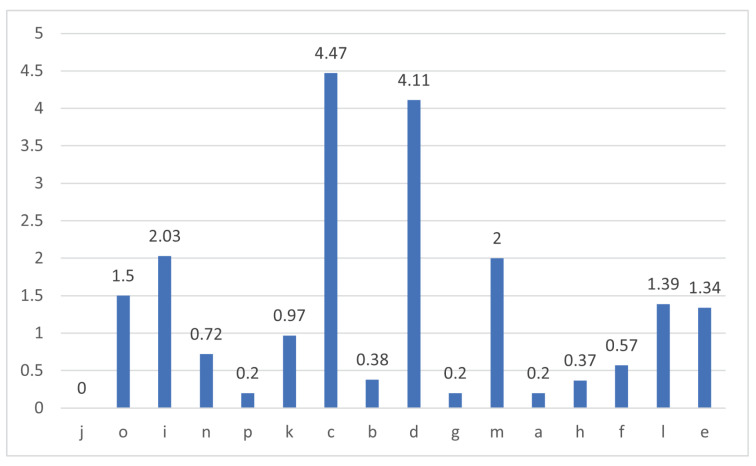
RSSI difference vs. reader–antenna position.

Figure 5 infers that an increase in distance between tag and antenna decreases the RSSI value. This inference was supported with OLS regression results in Table 6 and metrics in Table 5. OLS regression results have a negative coefficient of Distance_1 and Distance_2 variables. Negative coefficients present an inverse relation with output variable RSSI. Metrics in Table 5 show the *j* position has the highest RSSI value 86.98, which is the closest reader–antenna position to the tag and the *e* position has the lowest RSSI value, which is the farthest reader–antenna position to the tag. All three sets of results presented in Figure 5 and Table 5 and Table 6 confirm that closer positions would have higher RSSI values. When considering the dimensions of the laboratory in this research, position *j* could be the first preference for reader–antenna. The probability of selecting two better positions for signal receivable would be narrowed down to 15 combinations as position *j* was considered best. The second position was decided based on the spread of reader–antenna positions in Figure 3 and each individual antenna RSSI value. With this, *j* and *e* combination would be good pair of positions in the laboratory to fix two UHF RFID reader–antennas. Other RSSI values presented in Figure 7 show that the difference of RSSI value from positions *k* and *c* is 4.47 but their distance from static tag is almost same, similar to that on positions *b* and *d*. This could be useful in identifying multiple tags that are associated with patients where their initial reference could be associated with their hospital bed located in the room.

In Table 6, the *p* value shows the significance of each independent variable in predicting output variable when the value is less than the significance level α value (0.05). Based on this, Distance_1 and Distance_2 variables are significant and the Antennas_Distance variable is not, as its *p* value is far greater than α value (0.05). The *r*-squared value is 0.626, which is considerably low. However, the OLS regression model is able to find the relationship between independent and dependent variables. An increase in the *r*-squared value would enhance dependent variable prediction accuracy of the linear Equation (7) introduced previously.

## 6. Conclusions

Study results were presented in the architecture design of a two tier framework for data collection and data modelling. The main outcome from this study was to present an understanding of how to determine the optimum positioning for two RFID reader–antennas that would receive the maximum RSSI signal from a passive RFID tag in a simulated hospital ward. A circular polarisation technique was chosen for this purpose as this ultimate goal of the research is to design a remote patient monitoring system for ambulatory psychiatric patients that are highly vulnerable for risk of self-harm and suicide. Although this technique limited the range within which a tag could be read, it suited this clinical purpose that was designed to provide an assisting technical solution for reduced nursing staff levels on evening and night duty shifts when this risk is at its highest.

Radiation pattern, and reader–antenna and tag orientation play a major role in tag readability. This study created a combined data set from arbitrarily chosen positions of a pair of reader–antennas to produce a linear equation that estimated the Received Signal Strength Indicator (RSSI) from a passive RFID tag. The Decision Tree machine learning algorithm was best at predicting RSSI using regression modelling with the data set collected for this study and produced good performance metrics, compared with Random Forest, XGBoost and an Ensemble Learning model combining all three algorithms.

The biggest challenge for this study was tag readability due to reader–antenna radiation pattern and polarisation. The UHF 870 RFID readers with integrated antenna were designed to read RFID tags within range of 5 m using a one directional radiation pattern. However, with circular polarisation the reader–antennas was not able to read tags with distance more than 1.5 m. Another limitation of this study was that the Ensemble Learning with weighted average method had a higher error rate compared to the individual machine learning models. This could possibly be related to the relatively uncomplicated study design that had a small number of variables and therefore did not require the compensatory capabilities of Ensemble Learning algorithms. The *k*-fold cross-validation that was implemented in XGBoost model also had a high error rate which could also have impact upon its performance to correctly predict the output variable.

The future direction for this study is to replicate this method with a dynamic RFID passive tag so that data can be collected relative to tag motion. Near-field coherent sensing (NCS) technology would then be introduced to read human vital signs and subtle motions. Machine learning algorithms would again be employed to analyse the data patterns and build generic models for the remote patient monitoring system.

## Figures and Tables

**Table 1:** Reader–antenna coordinates in laboratory room/simulated ward.

Reader-Antenna Position	*x*	*y*	*z*
*a*	0	0	0.81
*b*	0	0.83	0.81
*c*	0	1.66	0.81
*d*	0	2.49	0.81
*e*	0	3.32	0.81
*f*	0.78	3.32	0.81
*g*	1.56	3.32	0.81
*h*	2.35	3.32	0.81
*i*	2.35	2.5	0.81
*j*	2.72	1.84	0.81
*k*	3.17	1.28	0.81
*l*	3.89	0.72	0.81
*m*	3.14	0	0.81
*n*	2.34	0	0.81
*o*	1.56	0	0.81
*p*	0.78	0	0.81
Tag	1.58	1.32	0.75

**Table 2:** Empirical experimental results of machine learning models in preliminary study.

Model	*r*-Squared	MSE
Decision Tree	1	0.01
Random Forest	0.99	0.40
XGBoost	0.97	1.04
Ada Boost Regressor	0.96	1.56
Gradient Boosting Regressor	0.95	1.87
Bagging Regressor	0.95	1.87
Extra Trees Regressor	0.94	2.31
SVR	0.67	12.46
NuSVR	0.65	13.39
Huber Regressor	0.63	14.28
Stochastic Gradient Descent	0.62	14.44
Linear Regression	0.62	14.44
Transformed Target Regressor	0.62	14.44
Lars	0.62	14.44
Orthogonal Matching Pursuit CV	0.62	14.59
RidgeCV	0.62	14.59
Ridge	0.62	14.59
RANSAC	0.62	14.59
Lasso-Lars IC	0.62	14.59
Poisson Regressor	0.62	14.66
Bayesian Ridge	0.62	14.66
LassoCV	0.62	14.66
ElasticNetCV	0.62	14.66
LarsCV	0.61	14.66
Lasso-Lars CV	0.61	14.66
Hist Gradient Boosting	0.6	15.28
LightGBM	0.59	15.52
kNN	0.58	15.92
Passive Aggressive Regressor	0.53	17.80
Lasso	0.53	17.97
Orthogonal Matching Pursuit	0.52	18.31
Elastic Net	0.46	20.70
Gamma Regressor	0.4	22.94
Generalised Linear Regressor	0.4	23.04
Tweedie Regressor	0.4	23.04

**Table 3:** Numerical variables and their statistical description.

	Distance_1	Frequency_1	RSSI_1	Distance_2	Frequency_2	RSSI_2
**count**	120.00	120.00	120.00	120.00	120.00	120.00
**mean**	1.91	921.43	73.39	1.74	921.79	77.21
**std**	0.35	2.05	5.19	0.39	1.69	7.02
**min**	1.25	918.80	66.53	1.25	918.80	66.53
**25%**	1.62	919.20	69.83	1.41	920.50	70.40
**50%**	1.98	922.40	72.50	1.59	921.90	81.56
**75%**	2.14	923.50	76.71	2.04	923.40	82.73
**max**	2.55	924.30	86.98	2.55	924.30	86.98

**Table 4:** Data set with top 5 records of features.

Antenna_1	Antenna_2	Distance_1	Distance_2	Antennas_Distance	Frequency	RSSI
*a*	*b*	2.06	1.66	0.83	920.5	76.71
*a*	*c*	2.06	1.62	1.66	920.5	77.09
*a*	*d*	2.06	1.97	2.49	919.2	72.60
*a*	*e*	2.06	2.55	3.32	920.5	70.20
*a*	*f*	2.06	2.15	3.41	920.5	70.20

**Table 5:** RSSI values for reader–antenna combinations.

	**a**	**b**	**c**	**d**	**e**	**f**	**g**	**h**	**i**	**j**	**k**	**l**	**m**	**n**	**o**
**a**															
**b**	76.71														
**c**	77.09	77.09													
**d**	72.6	76.71	77.09												
**e**	70.2	76.71	77.09	72.6											
**f**	70.2	76.71	77.09	72.6	69.26										
**g**	72.4	76.71	77.09	72.6	72.4	72.4									
**h**	70.2	76.71	77.09	72.6	69.83	69.83	72.4								
**i**	72.6	76.71	77.09	72.6	72.6	72.6	72.6	72.6							
**j**	86.98	86.98	86.98	86.98	86.98	86.98	86.98	86.98	86.98						
**k**	81.56	81.56	81.56	81.56	81.56	81.56	81.56	81.56	81.56	86.98					
**l**	70.2	76.71	77.09	72.6	67.87	69.26	72.4	69.83	72.6	86.98	81.56				
**m**	70.4	76.71	77.09	72.6	70.4	70.4	72.4	70.4	72.6	86.98	81.56	70.4			
**n**	82.73	82.73	82.73	82.73	82.73	82.73	82.73	82.73	82.73	86.98	82.73	82.73	82.73		
**o**	85.48	85.48	85.48	85.48	85.48	85.48	85.48	85.48	85.48	86.98	85.48	85.48	85.48	85.48	
**p**	82.53	82.53	82.53	82.53	82.53	82.53	82.53	82.53	82.53	86.98	82.53	82.53	82.53	82.73	85.48

**Table 6:** Ordinary least squares (OLS) regression results.

	*Coef*	*Std Error*	*t*	p>|t|	[0.025	0.975]
Const	109.2178	2.427	45.010	0.000	104.412	114.024
Distance_1	−6.0125	1.007	−5.972	0.000	−8.007	−4.019
Distance_2	−11.0570	0.874	−12.657	0.000	−12.787	−9.327
Antenna_Distance	0.2726	0.342	0.797	0.427	−0.405	0.950

**Table 7:** Performance metrics of regression models.

Model	MAE	MSE
Decision Tree	0.01	0.003
Random Forest	0.16	0.11
XGBoost	0.24	0.21
Ensemble Learning	0.04	0.006

## Data Availability

Not applicable.
